# Transcriptome analysis reveals novel patterning and pigmentation genes underlying *Heliconius* butterfly wing pattern variation

**DOI:** 10.1186/1471-2164-13-288

**Published:** 2012-06-29

**Authors:** Heather M Hines, Riccardo Papa, Mayte Ruiz, Alexie Papanicolaou, Charles Wang, H Frederik Nijhout, W Owen McMillan, Robert D Reed

**Affiliations:** 1 Department of Genetics, North Carolina State University, Raleigh, NC, 27695, USA; 2 Department of Biology, Pennsylvania State University, University Park, PA, 16802, USA; 3 Department of Ecology and Evolutionary Biology, University of California, Irvine, CA, 92697, USA; 4 Department of Biology and Center for Applied Tropical Ecology and Conservation, University of Puerto Rico, Rio Piedras, San Juan, Puerto Rico, 00921; 5 CSIRO Ecosystem Sciences, GPO 1700, Canberra, ACT, 2601, Australia; 6 Functional Genomics Core, Beckman Research Institute, City of Hope, Duarte, CA, 91010, USA; 7 Department of Biology, Duke University, Durham, NC, 27708, USA; 8 Smithsonian Tropical Research Institute, Panama City, Panama; 9 Department of Ecology and Evolutionary Biology, Cornell University, Ithaca, NY, 14853, USA

**Keywords:** *Heliconius*, Adaptation, Pigmentation, Ommochrome, Melanin, Genomics, Evo-devo

## Abstract

**Background:**

*Heliconius* butterfly wing pattern diversity offers a unique opportunity to investigate how natural genetic variation can drive the evolution of complex adaptive phenotypes. Positional cloning and candidate gene studies have identified a handful of regulatory and pigmentation genes implicated in *Heliconius* wing pattern variation, but little is known about the greater developmental networks within which these genes interact to pattern a wing. Here we took a large-scale transcriptomic approach to identify the network of genes involved in *Heliconius* wing pattern development and variation. This included applying over 140 transcriptome microarrays to assay gene expression in dissected wing pattern elements across a range of developmental stages and wing pattern morphs of *Heliconius erato*.

**Results:**

We identified a number of putative early prepattern genes with color-pattern related expression domains. We also identified 51 genes differentially expressed in association with natural color pattern variation. Of these, the previously identified color pattern “switch gene” *optix* was recovered as the first transcript to show color-specific differential expression. Most differentially expressed genes were transcribed late in pupal development and have roles in cuticle formation or pigment synthesis. These include previously undescribed transporter genes associated with ommochrome pigmentation. Furthermore, we observed upregulation of melanin-repressing genes such as *ebony* and *Dat1* in non-melanic patterns.

**Conclusions:**

This study identifies many new genes implicated in butterfly wing pattern development and provides a glimpse into the number and types of genes affected by variation in genes that drive color pattern evolution.

## Background

Recent advances in genomics have catalyzed the discovery of genes underlying adaptive phenotypic variation in non-model organisms [1-3]. These discoveries have yielded important insights into the genetic basis of phenotypic evolution, from understanding how genetic interactions and gene architecture may bias and constrain evolution [e.g., [4-9]] to how cooption and modification of gene networks may underlie novel phenotypes [e.g., [10-12]]. Phenotypic adaptation frequently occurs through variation at genes that potentially regulate a large number of downstream genes [8]. In non-model systems, little is known about the downstream elements these genes effect, making it difficult to surmise why such genes are selected to drive variation. Genome and transcriptome forward approaches facilitate discovery of multiple components of these gene networks, from the genes regulating these “switch” genes, to the cascading sets of genetic changes that follow from such genes of major effect.

Here we initiate investigation into the gene networks underlying the development and variation of adaptive wing patterns in *Heliconius* butterflies. This genus has long been a popular system for studying the genetics underlying phenotypic diversification [13-15]. *Heliconius* exhibits extensive wing color pattern variation across its ~40 constituent species. In almost all cases this diversity is driven by Müllerian mimicry, which allows local populations of noxious species to enhance their ability to deter predators through shared warning coloration. The species *Heliconius erato* and *Heliconius melpomene* are particularly remarkable in their intraspecific color pattern variation, as they converge on over 20 mimetic wing patterns in various regions of the neotropics [16-18]. These phenotype-rich and highly convergent species provide an opportunity to study how complex variation in developmental patterning networks can arise within species and diversify under natural selection.

**Figure 1:**
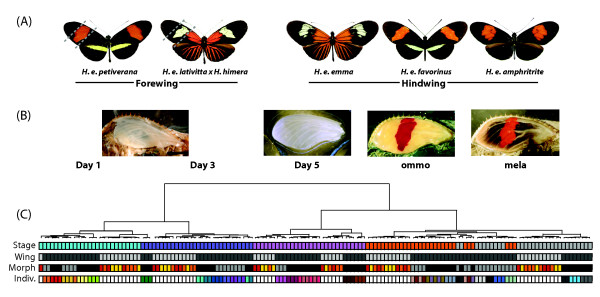
***Heliconius* butterflies sampled and sources of genetic variation in gene expression.** (**A**) Color pattern morphs sampled for each wing. The *optix* gene controls two general alternative phenotypes: 1) a forewing with a red medial band and a non-red hindwing, and 2) a forewing with red base and a hindwing with red rays. Forewings were sampled in three sections as indicated and hindwings were sampled whole. Each color pattern is comprised of a mosaic of pigmented scale cells: red scales bear ommochromes, yellow scales bear 3-OH-kynurenine, and black scales bear melanin. (**B**) Appearance of the forewing at each of the 5 sampled developmental stages, illustrating the sequence of scale maturation and pigment deposition. (**C**) Array results clustered by similarity in transcription. Colored blocks represent the phenotype of each array sample, including Stage (the stage of development sampled, matching to the aligned stages in part B), Wing (hindwing: light gray; forewing: dark grey), Morph (*amphitrite*: red; *emma*: orange; *favorinus*: yellow; hybrid form: black; *petiverana*: grey), and for forewings, by Individual, highlighting the relationships among the three dissected wing sections per individual (each individual is represented by a single color, with color arbitrarily chosen). The majority of expression variation is associated with developmental stage, with further clustering by Wing, Morph, and Individual.

Significant progress has recently been made in understanding the genetic basis of color pattern diversity in *Heliconius*. Genetic mapping has shown that much of the color pattern variation across the genus is attributable to natural variation at only three loci of major effect [19-21]. In *H. erato* and *H. melpomene*, these three genomic intervals control several distinct color patterns, including the red color pattern elements (variation in the red forewing patches and hindwing rays), the presence of a yellow hindwing bar, and variation in complex black patterns on the forewing (Figure 1) [17,22-25]. These loci ultimately regulate scale-level differences in pigmentation, turning on the tryptophan-ommochrome pathway to impart red (ommochromes) and yellow (3-OH-kynurenine) coloration, and the melanin pathway for black coloration [26].

Of the three major color pattern loci, most is known about the one that controls red color patterns. At this locus, the gene controlling red pattern variation has been identified as a homeobox transcription factor called *optix*[ 27]. The patterning role of *optix* is particularly well illustrated by how its spatial expression patterns foreshadow the future location of red color patterns across diverse *Heliconius* species. This differential expression, coupled with a lack of amino acid variation in the optix protein, indicates that red pattern variation is a result of *cis-*regulatory variation between *optix* alleles [18,27]. *optix* is best known for its role in eye development [28], leading to the suggestion that *optix* may be turning on gene networks leading to the eye-associated ommochrome pigments in the wings [12,27]. One of the main challenges we now face for understanding the evolution of *Heliconius* wing patterns is to uncover how changes in *optix cis*-regulatory elements (CREs) produce such a wide array of complex red color patterns within and between *Heliconius* species. It is unknown what developmental prepatterns drive *optix* expression, how allelic variation in *optix* CREs responds to these prepatterns, or what downstream genes *optix* regulates to control pigmentation.

In this study, we take a transcriptomic approach to begin to piece together the gene networks that act upstream and downstream of *optix*. We undertook a large series of microarray experiments to analyze transcription across multiple wing tissues, developmental stages, and color patterns morphs of *H. erato*. Our analysis targeted two main classes of genes: 1) upstream regulatory genes that may be spatially regulating *optix* expression, and 2) genes differentially activated downstream of *optix* to play a role in the differentiation of pigment-bearing scale cells. To determine candidates for upstream regulators of *optix,* we looked for transcripts expressed differently across proximal to distal sections of the forewing prior to *optix* expression. Because *optix* is a transcription factor that responds to pre-existing positional information, it can be inferred that a butterfly from any given race should express the full repertoire of regulatory positional information to produce any of the *optix*-related wing patterns. Thus, there must be a common, conserved regulatory prepattern that different *cis*-regulatory alleles of *optix* interpret in different ways. Since this prepattern should be the same across all *H. erato* races, screening for genes differentially expressed between color pattern morphs would not be useful for identifying transcripts for prepatterning genes. Given this, we sought to look for transcripts whose expression was consistently associated with proximal, medial, and distal wing sections dissected along color pattern boundaries. Conversely, to assess how *optix* regulates downstream gene expression to specify scale phenotypes we looked for transcripts with differential expression among differently colored wing pattern elements of both the forewing and hindwing. Our results provide several strong candidates for regulators of *optix* and reveal a number of structural and pigmentation genes correlated with specific color pattern elements. These data allow us to begin to understand the function of *optix* in terms of a wider network of patterning and pigmentation genes and bring us closer to understanding the developmental genetic architecture of color pattern evolution in *Heliconius.*

## Methods

### Butterfly stocks and sample preparation

This study is an analysis of two distinct datasets generated using the same microarray platform. One dataset involved comparative analysis of forewing sections of different color morphs, while the other compared whole hindwings with different color patterns.

For the forewing analysis we compared proximal, medial, and distal wing sections of two color pattern morphs: *H. erato petiverana* and a hybrid *H. himera* x *H. erato etylus* (Figure 1). This hybrid stock was generated to ensure that a comparable wing section dissected from the two morphs contained a single unique color pattern element (races for the hindwing study vary in the extent of black on the mid-forewing section). The proximal section in *H. erato petiverana* is black and the hybrid form orange/red, while the medial section is red in *H. erato petiverana* and pale yellow in the hybrid form. The distal section is black in both races and thus acts as both a control for potential morph-specific effects unrelated to color and a contrast for tissue-specific effects. *H. e. petiverana* stocks were developed from Panamanian individuals and the hybrid *H. himera* and *H. e. etylus* stocks were generated from Ecuadorian individuals that were crossed and selected across several generations to create a stock with a consistently homozygous phenotype [27].

For the hindwing analysis, we compared hindwing color pattern gene expression in three races that meet in a hybrid zone in Peru. *H. erato emma* has a rayed hindwing, *H. erato favorinus* has a yellow-barred hindwing, and *H. erato amphitrite* has a black hindwing (Figure 1). Because alleles are thought to flow freely across these hybrid zones, with exception of color pattern genes, we should expect these populations to be similar genetically aside from color pattern-related differences [29], making population controls less necessary. Stocks of each of these species were developed from individuals collected from natural populations.

Specimens were reared from stocks maintained at the STRI *Heliconius* Rearing Facility in Gamboa, Panama. Each larva was reared separately on *Passiflora* hostplant and monitored for the time of pupation. Wings were dissected at five time intervals: 1, 3, and 5 days after pupation, when orange/red ommochrome pigments were beginning to be expressed (~7 days after pupation), and when black melanin pigments were starting to pepper the center of the wings (~8 days after pupation). Pupae for forewings were reared at ambient temperature (~28°C), while pupae for hindwings were kept indoors at 25°C. In the forewings, Days 1, 3, and 5 were at 12, 36, and 60 h post-pupation. In the hindwings these stages were sampled at 24, 48, and 72 h. Although potentially yielding differences, the discrepancy between the temperature and timing should put each stage closer to the same developmental stage. Wings were dissected from chilled pupae and stored in RNAlater® (Ambion) at −20°C. Forewings were cut into proximal, medial, and distal sections during dissection using wing venation landmarks as a guide.

### Microarrays

Samples hybridized to microarrays included three replicates each of each race, stage, and wing section for forewings (3 replicates × 2 morphs × 3 wing sections × 5 stages, with one replicate wing missing for Day 1 *H. e. petiverana* = 87 samples) and four replicates of each stage and race for hindwings (4 replicates × 3 races × 5 stages = 60 samples). RNA extraction and amplification protocols used are outlined in [27]. Cy3-labeling of samples, hybridization, and array scanning was performed according to NimbleGen protocols (2008): for the forewings this was performed at the City of Hope Functional Genomics Core, while the hindwings were run separately at NCSU.

Samples were hybridized to NimbleGen HD2 12-plex arrays. These arrays include 12 identical subarrays with 135,000 60 bp probes each, each hybridizing a separate sample. Samples were distributed across arrays to prevent repeat conditions as much as possible and to space similar conditions in different regions of the slide. The array design involved two classes of probes. First there was a tiling component involving 89,310 probes tiled across three genomic intervals. Results from the tiling data were used for the initial discovery of the *optix* gene [27] and are not the focus of the present study. The second component involved a representation of a set of 12,450 transcript contigs at 1-6X coverage for a total of 40,046 probes, with a mean coverage of 3–4 probes per contig. The number of probes for each contig depended on the ability to create suitable probes according to NimbleGen probe selection criteria and was limited by the small size of some transcripts and the minimum spacing criterion of 15 bp apart. Sequences of low complexity and high repeats with the rest of the genome (>5X representation), determined by comparison against 1.6 MB of genomic sequence available at the time, were avoided for designing probes. An additional 3,248 random probes were placed on the array for quality control.

The transcriptome data used for the array design includes an assembly of two data sources: 1) Sanger EST data from a mixed species and race library built from pooled RNA from *H. erato petiveranaH. erato eratoH. erato cyrbia*, and *H. himera* fore- and hindwing tissues extracted from 5th instar larvae, prepupae, Day 1 pupae, precolor pupae, and 10 days post-pupation and 2) 454 EST data from the same races and stages used for the forewing study here. The assembly was produced using the MIRA3 [30] assembler via the est2assembly [31] assembler parameterization & annotation package. To facilitate microarray probe design, assembled sequences were randomly resolved of polymorphisms and large regions of polymorphic sequence were treated as missing (0.6% of the transcriptome data was polymorphic; 0.7% of sequence data was treated as missing). SNP variation among races is expected to be relatively low: in a sampling of both coding and non-coding genomic sequences of 45 *H. erato* individuals from 8 races, 1.7–2.6% of called sequence positions were variable per individual relative to a *H. e. petiverana* reference (Supple et al., unpublished). All transcripts from this assembly shorter than 200 bp were excluded to avoid false assembly and allow multiple optimal probes. Although these transcripts may not exhaustively represent the entire transcriptome, they should represent a majority of the genes expressed at moderate levels during pupal wing development. The transcriptome assembly used for the microarray is available at InsectaCentral [32] (IC33431).

### Data analysis

As an initial quality control measure we examined the distribution of probe intensities across each array. Regions with uneven intensities were removed from the dataset and treated as missing data. Arrays with large regions of uneven intensities or with inconsistent intensity distributions from other samples were rerun. To examine the effects of normalization, we used hierarchical clustering and principal components analyses to compare array clustering across raw and normalized datasets and examined the influence on distribution of intensities of all probes and random probes. Quality control procedures and microarray data analyses were performed using JMP Genomics (SAS Institute). Forewing and hindwing datasets were normalized separately, except for the combined color ANOVA, where they were normalized together. Similar intensity distributions across arrays of both studies prevented normalization methods from having a strong impact on intensities. Data were log_2_ transformed and Loess normalized using all transcript probes. Expression for each transcript was then summarized as a mean of the probes representing it.

We performed ANOVAs to identify genes that were differentially expressed for phenotypic comparisons of interest. For the proximal-distal wing section analysis we used the forewing data to perform an ANOVA by wing section, with Stage, Wing Section, and Stage*Wing Section as fixed effects and Morph as a random effect. We retained significant genes passing an FDR of 0.01, including only comparisons of wing sections within stage.

To identify genes differentially expressed in association with color phenotype we first used a color-specific ANOVA on the combined forewing and hindwing dataset, with conditions black, red, and yellow. Hindwings were classified as one color, with each race a different color (Figure 1), although the red and yellow contain a large portion of the wing that is black. This analysis included Color, Stage, and Color*Stage as fixed effects with significant comparisons by color within stage with an FDR of 0.01 kept for further analysis. In case the color dilution from black regions of the yellow and red hindwings influenced the results, we also performed forewing-only ANOVAs by Color, one treating the single yellow forewing section as black and the other treating this section as red, with Morph as a random effect.

We wanted to ensure we were not missing any color-specific genes due to interactions of morph, wing section, and wing-specific effects, therefore we also applied data filtering methods to all significant genes from a series of pairwise comparisons from ANOVAs. For this we performed pairwise tests on each wing separately, comparing each gene across forewing (proximal, medial, and distal for each morph) and hindwing (each of the three races) tissues within each stage. For the forewing analysis this involved Morph, Stage, Wing Section, and Morph*Stage*Wing Section as fixed effects and Slide as a random effect. In the hindwing this involved Morph, Stage, and Morph*Stage with Slide as a random effect. We retained significant transcripts from any pairwise comparisons using a threshold FDR of 0.01, considering only morph comparisons within stage for hindwings and, for forewings, within stage and either between wing sections within a morph or between the same wing section between morphs. Because several genes were significant in ways inconsistent with color phenotype (e.g.*,* differentially expressed across whole forewings between morphs or along the proximal-distal axis), we performed a p-value filtering procedure on these transcripts that isolated the genes whose differential expression patterns were most consistent with color differences. This involved a Fisher’s method for p-value summation (−2∑ln(p)) on all comparisons that are consistent with red areas having different expression than non-red areas (red and black hindwings, red and yellow hindwings, the hybrid proximal and medial; hybrid proximal and distal; *H. e. petiverana* proximal and medial; *H. e. petiverana* medial and distal; hybrid and *H. e. petiverana* proximal; hybrid and *H. e. petiverana* medial) and excluding comparisons between yellow and black or between black sections. In this summation we set a cap on the low end of p-values of 0.0001, which is close to the global FDR threshold, to avoid overly highly significant patterns from any one stage dominating the summed value. The final summed p-value was ranked and all summed values greater than the value obtained if p<0.01 for each comparison were retained (~10% of the genes). These were then combined with all significant genes that are differentially expressed by color from the color-based ANOVA. As an additional filter, we subsequently performed modulated modularity clustering [33], a method that clustered together genes with similar expression patterns using mean expression of genes for each condition. The resulting modules were used to remove genes that were yet inconsistent with expectations based on color phenotypes.

### Annotation and functional enrichment analysis

All transcripts were assigned gene identifications using two different procedures. First, transcripts were blasted against FlyBase genes [34] using tblastx and the top hit for each transcript satisfying an E-value of 1E-5 or less was kept. Second, all transcripts were run through Blast2Go [35] blastx against the Genbank non-redundant protein database with a 1E-5 threshold for keeping hits. In Blast2Go, all hits above this threshold were used for identification and annotation term assignment. Of the 12,450 transcripts on the array, ~35% (35% Blast2Go; 33% Flybase) of genes had blast hits to functional annotations in other insect genomes and ~5% (7% Blast2Go, 4% Flybase) had hits with no functional annotation. The large fraction of transcripts with no hits appeared to be mostly due to transcript contigs from highly divergent UTR regions; examination of a number of these transcripts revealed that they are non-coding and map to the ends of genes from the *H. melpomene* genome [36].

Color-specific and proximal-distal gene lists were annotated further. This involved combining transcripts into unique genes using several factors including 1) blast hits to the same FlyBase or Blast2Go gene, 2) hits to the same part of the *H. melpomene* genome [36], and 3) assignment to the same modular cluster [33] and/or highly similar expression profile across development and conditions. In the case where transcripts had no hits satisfying our thresholds, we improved gene identification using longer versions of the genes containing these transcripts found using blastn to other Lepidopteran transcriptomes available in ButterflyBase [37] or using the *H. melpomene* genome [36]. All of these methods required ultimately blasting to another organism with a gene ID with E-value <1E-5.

To examine gene function and potential enrichment of certain functions in our gene lists we used two separate programs. First, we used a *Drosophila*-specific gene annotation enrichment analysis in DAVID [38,39] using the top FlyBase hits for each gene. Analyses included examining both enriched terms and clusters of similar terms. We consider this analysis to more accurately portray functionality as functions are more insect specific and, in cases where two transcripts blasted to the same FlyBase gene, these were treated only as a single instance, thus reducing the effect of error in response to multiple contigs of each gene being represented. We also performed a Blast2Go functional enrichment analysis. This involved acquiring annotation GO terms from blastx to each transcript, blasting for additional protein functional terms using InterProScan, and augmenting the annotation using the annex function. Background gene lists for enrichment analyses included all genes on the array. This is justified given that very few genes were not expressed in all samples. We tested for functional enrichment among differentially expressed transcripts of the forewing, hindwing, forewing and hindwing combined, color-specific genes, and proximal-distal section genes. In addition to specific functional clusters, we also examined the significance of genes thought to interact with *optix* in *D. melanogaster* retinal development [40], brain development [41], and embryogenesis [42] ( Additional file 1).

Raw and normalized data files and experimental design files are available at the NCBI Gene Expression Omnibus (GSE38084). We have also availed a file on Dryad (doi:10.5061/dryad.f76f3) including 1) mean values for each condition and mean probe values for each sample, 2) identification terms for the various sources of annotation (Blast2Go, Drosophila-based, manual), and 3) significance of each transcript across the variety of tests and filters (e.g., hindwing, ANOVA color, proximal-distal forewing ANOVA).

## Results

### General transcription patterns

Hierarchical clustering and principal component analyses of gene expression across microarray samples revealed that most of the variation in gene expression was associated with developmental staging (Figure 1C). Stage-specific clusters were related largely in concordance with developmental time, with Day 1 and Day 3 forming sister clusters and Day 5 samples clustering closer to ommochrome-stage and melanin-stage samples. Unlike the other stages, ommochrome- and melanin-stage arrays were more intermixed in their expression similarities, with the hybrid forewing melanin phenotype clustering more closely with the hybrid ommochrome phenotype rather than with the rest of the melanin samples. This could be explained by the more extreme genetic differences within this hybrid form and the closer developmental timing between ommochrome- and melanin-stages. Variation in expression between arrays was subsequently clustered by wing, forewings were further separated by morph, and sections of forewings from the same individual tended to cluster within morph (Figure 1C). Thus, comparatively few genes differed by wing section in the forewings or by morph within hindwing comparisons.

### Transcription associated with proximal-distal patterning

In the forewing analysis we identified 338 transcript contigs differentially expressed in a manner consistent with proximal-distal expression differences. These contigs were found to represent 215 unique transcripts, 152 of which corresponded to genes with functional annotations ( Additional file 2).

**Figure 2:**
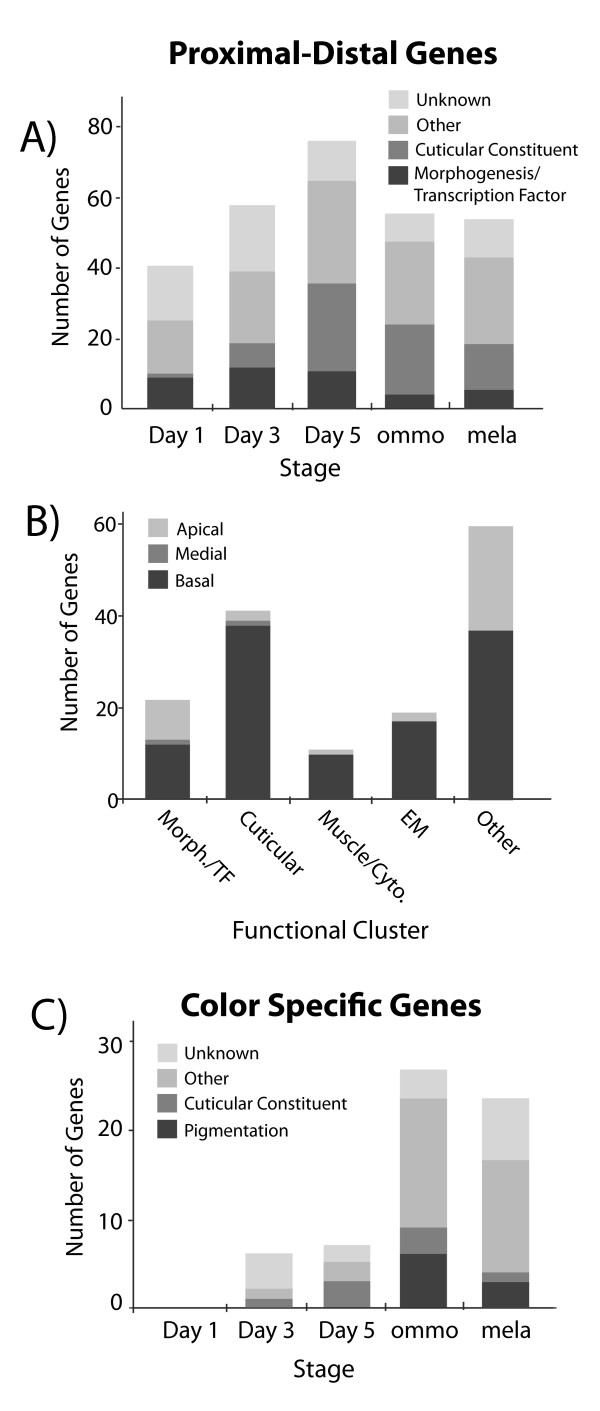
**Number and function of differentially expressed genes by stage.** This includes genes that vary along the proximal-distal axis (**A**, **B**) and genes differentially expressed by colored wing section (**C**). Apical, Medial, and Basal refer to sections of the forewing. Functional categories are defined by inference from gene ontology functional clustering in DAVID.

**Figure 3:**
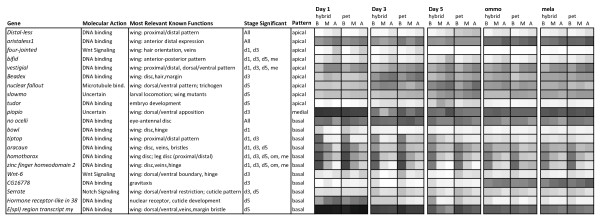
**Transcription and morphogenesis factors with significantly variable expression across the proximal-distal axis.** Gene names and relevant known functions are largely based on information and gene ontology categorization available in FlyBase. “Pattern” defines whether expression is highest apically or basally or whether variation separates the medial from peripheral regions. The heat map shows the mean intensity of expression across the conditions of the forewing arrays, with darker shading indicating more intense expression and levels relative to all gene intensities. Stages of differential expression are abbreviations of stages presented in Figure 1. pet: *petiverana*; B: basal; M: medial; A: apical.

The functional distribution of the proximal-distal genes was dominated by five main classes of enriched gene functions: cuticle proteins, extracellular matrix genes, morphogenesis and transcription factors, immune-related genes, and muscle and cytoskeleton related genes (Figure 2A,B, Table 1, Additional file 2). The majority of functionally annotated proximal-distal genes showed higher expression proximally than distally (Figure 2B, Additional file 2). The more structurally related genes (muscle, cytoskeletal, cuticular, and extracellular matrix) were almost exclusively higher in proximal expression, while the morphogenesis and transcription factors had nearly equal representation of proximal and distal genes. Morphogenesis and transcription factor genes were more abundant earlier in pupal development from Day 1 – Day 5 (Figure 2A), and included several genes known for their roles in imaginal disc wing and appendage development and wing axis formation (Figure 3). Several of these genes had long-term persistence in proximal-distal expression across development (Figure 3). In later stages (Day 5+) the proximal-distal genes were dominated by cuticle proteins (Figure 2A).

**Table 1:** Enriched functional gene clusters for each list of significant genes inferred using DAVID

**Functional enrichment clusters**			
**All significant genes**	*Score*	**Forewing**	*Score*
structural constituent of cuticle	9.61	structural constituent of cuticle	9.19
chitin binding	2.43	cytoskeleton/muscle	1.26
pigmentation	1.44	redox activity	1.21
aging	1.41	Immunoglobulin-like	1.17
muscle related	1.16	oxidative response	1.03
thioredoxin; oxidoreductase	1.16	ribosomal	1.02
ATP nucleotide binding	1.15	motor activity	0.93
metal binding	0.89	Aging, response to oxidative stress	0.93
oxidative response	0.88		
hormone receptor	0.87	**Hindwing**	
growth	0.84	structural constituent of cuticle	2.23
cellular localization	0.83	chitin binding	2.04
homedomain related	0.80	pigmentation	1.94
GTP related	0.71	visual perception and cognition	0.78
cytoskeleton	0.71	metal binding	0.78
intrinsic to plasma membrane	0.70	intrinsic to transmembrane	0.58
hormone and odorant binding	0.69	GTP related	0.55
immune response	0.67		
carboxypeptidase activity	0.65	**Proximal-Distal**	
motor activity	0.67	structural constituent of cuticle	9.55
serine-type peptidase activity	0.59	extracellular matrix and cell adhesion	2.95
biogenic amine metabolic process	0.55	morphogenesis/transcription factors	2.23
		immunoglobulin-like	1.61
**Color-Specific**		chitin binding	1.29
structural constituent of cuticle	1.96	cytoskeleton/muscle	1.16
		notch signaling pathway	1.05
		zinc-finger and ion binding	0.72

Category names are a summary of all gene ontology terms assigned to a functional gene cluster using the gene functional classification tool in DAVID. Only functional classes with scores greater than 0.50 are displayed.

### Transcription associated with color pattern variation

1,784 transcripts were differentially expressed in pairwise comparisons of tissues across forewings (1298) and hindwings (479). Of these genes, 206 crossed our p-value threshold to be considered as color-specific expression. Remaining genes not assigned to color-specific or proximal-distal patterns (Figure 2) tended to involve either a single forewing section of one race that showed differential expression, differential expression observed in the hindwing only, or morph-specific effects independent of forewing tissue section. A color-specific ANOVA recovered 242 genes, of which 60 overlapped with the pairwise comparison p-value list. After further filtering for color-specific expression using modularity clustering, only 72 transcripts from this combined list were considering related to final color phenotype. These transcripts represented 51 unique genes, with 38 having hits to known proteins (Table 2, Additional file 3).

**Table 2:** Genes differentially expressed by colored wing region

**Stage**	**Gene**	**Red devo pattern**
**Day 3**	optix	upregulated
	cuticular protein 67B (Cpr67b)	downregulated
	Uncharacterized (CG8483)	upregulated
	Unknown (IC33431AfEcon3556)	upregulated
	Unknown (IC33431AfEcon6355)	DR upregulated
	Unknown (IC33431AfEcon8390)	upregulated
**Day 5**	optix	upregulated
	cuticular protein 66Cb [2]	upregulated
	cuticular protein 66Cb [1]	DR upregulated
	Cuticular protein 100A	DR on longer
	alpha-N-acetylgalactosaminidase (CG5731)	DR downregulated
	Unknown (IC33431AfEcon11770)	DR downregulated
	Unknown (IC33431AfEcon12188)	nonred upregulated
**ommo**	optix	upregulated
	**Kynurenine formamidase 1 (kf1)**	on earlier
	**ATP-binding cassette transporter-C (BmABC007735)**	upreg./on earlier
	**ATP-binding cassette transporter-C (BmABC007785)**	upregulated
	**B2G: monocarboxylate transporter (CG8034)**	on longer
	Adult cuticle protein 65Aa [1]	FW upregulated
	B2G: organic cation transporter (CG7458)	on earlier
	B2G: pap-inositol-phosphatase (CG7789)	upregulated
	tousled-like kinase	on earlier
	B2G: exosome component 10-like	on earlier
	Adult cuticle protein 65Aa [2]	FW upregulated
	Adult cuticle protein 65Aa [3]	FW upregulated
	B2G: rag-1 activating protein (CG7272)	upregulated
	B2G: glycoside hydrolase (CG9701)	upregulated
	B2G: wing disc specific protein	upregulated
	Unknown (IC33431AfEcon12235)	upregulated
	Unknown (IC33431AfEcon7324)	upregulated
**ommo**	**cinnabar**	upregulated
**+mela**	**yellow-d**	upregulated
	B2G: estrogen sulfotransferase (CG6704)	upregulated
	B2G: synaptic vesicle protein (CG31106)	up reg./on longer
	B2G: alkaline phosphatase (CG5150)	upregulated
	waterwitch	upregulated
	Paps sythesase (Papss)	upregulated
	superoxide dismutase containing protein (CG31028)	upregulated
	Unknown (IC33431AfEcon5187)	FW upregulated
	Unknown (IC33431AfEcon10072)	upregulated
**mela**	**ebony**	upreg./on longer
	Nicotinamide amidase	upregulated
	B2G: chitinase	upreg./on longer
	Glucose transporter 1	on longer
	Glucosyltransferase 1 (GlcT-1)	upregulated
	B2G: organic cation transporter (CG6126)	upregulated
	B2G: carboxylcholinesterase (CG6018)	upregulated
	Odorant binding protein 56a	upregulated
	Uncharacterized (CG9628)	on longer
	Unknown (IC33431AfEcon2028)	upregulated
	Unknown (IC33431AfEcon4212)	upregulated
	Unknown (IC33431AfEcon5741)	upregulated
	Unknown (IC33431AfEcon7642)	upregulated

Names are from FlyBase except where B2G is indicated, in which case genes lacked FlyBase names so Blast2Go gene names were used, and for the ABC transporters, where *Bombyx* IDs were used [43]. Known pigment genes are designated by black bold (melanin genes, putative ommochrome genes shown in Figure 5). Transcripts with no hits to annotated genes are indicated with their transcript IDs. *DR* = dennis (basal red forewing patch) and rayed differentially expressed only. *FW* = forewing.

**Figure 4:**
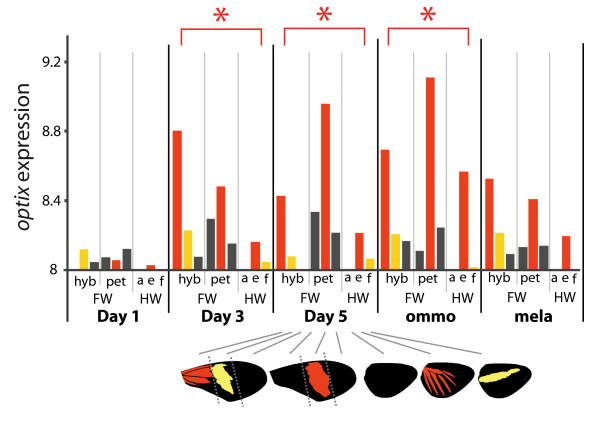
**Mean gene expression of *optix* by wing section.** Bars are colored by the distinguishing color of the wing section. Asterisks indicate stages with significant differential expression by color. Y-axis gene expression is log_2_ microarray intensities. hyb: hybrid form; pet: *petiverana*; a: *amphitrite*; e: *emma*; f: *favorinus*; FW: forewing; HW: hindwing.

Among the unique genes, none showed pattern-specific expression differences in Day 1. *optix* was the first gene observed to be differentially expressed in a consistent red-specific manner (Table 2), beginning at Day 3 (Figure 4). *optix* maintained pattern-specific transcription longer than any other gene, extending into the late stages of pigment development. Beyond *optix*, there were few genes that show pattern-related expression at Day 3 and Day 5. Those that did were mostly cuticle proteins with uncertain color affinities, as they did not have the straightforward red-related expression across wing color comparisons displayed by *optix*.

**Figure 5:**
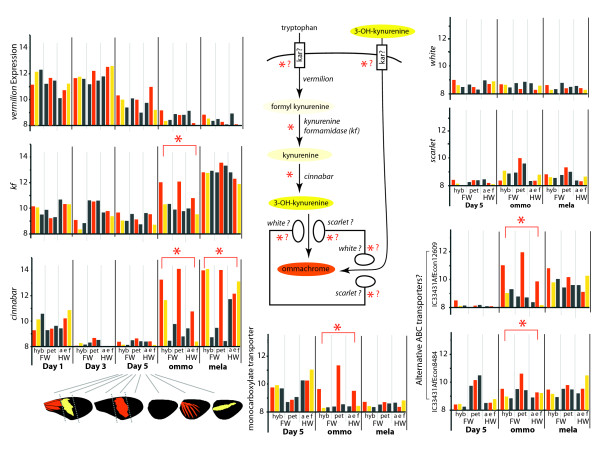
**Mean gene expression levels for the major genes in the ommochrome pathway.** The putative ommochrome pathway based on *D. melanogaster* and derived from Reed et al. [59] is inset. In this pathway tryptophan or external 3-OH-kynurenine may enter the cell through a transporter, tryptophan is enzymatically processed to the 3-OH-kynurenine precursor, and this precursor is hypothesized to be shunted into a pigment granule potentially involving eye ommochrome transporters *white* and *scarlet*. Molecules imparting orange/red and yellow pigmentation are indicated in color. Asterisks indicate stages with significant differential expression in the charts and enzymes with differential expression in the pathway. white, scarlet, and potentially karmoisin are the transporters identified from *D. melanogaster* eyes and play an uncertain role in the butterfly ommochrome pathway. We propose alternative transporters may be involved (monocarboxylate transporter, alternative ABC transporters). Y-axis gene expression is in log_2_ microarray intensities. Abbreviations and other style follows Figure 4.

**Figure 6:**
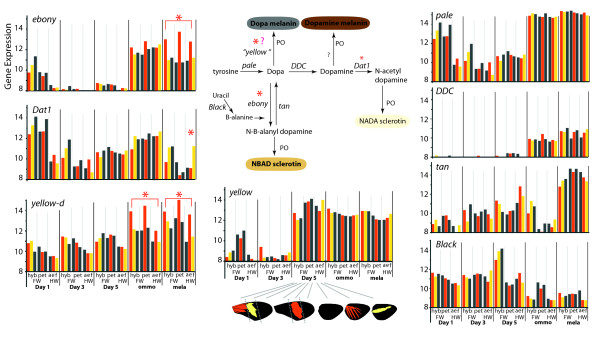
**Mean gene expression levels for the major genes in the melanin pathway.** The insect melanin pathway, inferred from work in *D. melanogaster* pathway [44], shows the major enzymes involved in insect melanization. Molecules in this pathway imparting final color differences are indicated with their respective colors. Asterisks indicate stages or pairwise comparisons with significant differential expression in the charts and enzymes with differential expression in the pathway. Y-axis gene expression is in log_2_ microarray intensities. Abbreviations and other style follows Figure 4.

The majority of color pattern-related expression differences were observed during the late stages of ommochrome and melanin pigment development (Table 2, Figure 2). Many of these transcripts showed expression differences spanning both pigmentation stages (Table 2, Additional file 3). All transcripts differentially expressed in the ommochrome- and melanin-stages had increased expression in red and/or yellow regions rather than in black regions (Figures 5 and 6, Table 2, Additional file 3).

Several color-specific transcripts could be assigned to the tryptophan-ommochrome biosynthesis pathway (Figure 5). Among these we found red-associated expression of both *kynurenine formamidase (kf)* and *cinnabar* – genes that encode enzymes required for ommochrome synthesis. We also identified three transporter genes previously undescribed for pigment transport - two ABC transporters and a monocarboxylate transporter - transcribed in strong association with red wing patterns. Our ABC transporter transcripts had clear matches to the *H. melpomene* genome, whose full gene sequences blasted distinctly to the ABC-C class of transporters defined for insects [43] (Table 2). Recognized ommochrome transporters *scarlet* and *white* are also ABC transporters, but, along with pigment transporter candidates *brown* and *atet-like*, they belong to the ABC-G transporter subclass [43]. *scarlet* and *white* showed very low expression levels and no association with color pattern.

Stage-specific expression of melanin synthesis genes was consistent with their presumed pigment and cuticle synthesis functions in late pupal development (Figure 6). *ebony, dopa decarboxylase (DDC),* and *pale* showed strong upregulation during the initial stages of ommochrome synthesis, a time when scale cells first begin to develop thick cuticle. *yellow* showed upregulation earlier at Day 5, while *tan* was upregulated during melanization. Regarding differential expression, *ebony* exhibited significantly higher expression in red versus black patterns during melanin synthesis. *yellow-d* was also upregulated in red patterns, but in both ommochrome and melanin stages. *Dopamine N-acetyltransferase 1* (*Dat1*) showed higher expression in yellow patterns during melanin synthesis, however this pattern was only significant in hindwings. Various other genes of uncertain function were differentially expressed in a color-specific manner (Table 2, Additional file 3).

### Functional enrichment analysis

Results from the DAVID and Blast2Go analyses were largely consistent in the functions disproportionately represented in gene lists (Table 1). In this analysis, for each gene list the most enriched gene function was structural constituent of the cuticle (Table 1), representing the diverse cuticle proteins that were significantly differentially expressed across all analyses. There were a few additional genes in the hindwing alone that were of interest. In particular, the significantly different genes among hindwings were enriched for pigmentation genes, including some not found to be different in the forewing, such as *lightlightoidDat1*, and *ovo*. Like *Dat1light* and *lightoid*, genes implicated in vesicular transport for pigment granule formation [45-47], were higher in the yellow-barred *H. e. favorinus* during the late stages of ommochrome or melanin pigment development, whereas *ovo* was higher in *H. e. emma* Day 3 ( Additional file 3). Of the 22 genes recognized to potentially interact with *optix* in *D. melanogaster*, seven were present in the array transcriptome, and of these, only *tiptop* and *homothorax* were differentially expressed along the proximal-distal axis ( Additional file 2).

## Discussion

Since Carroll et al. [48] described the first gene expression patterns associated with butterfly wing pattern development, researchers have used the candidate gene approach to identify over a dozen developmental genes associated with eyespot and stripe pattern development in various butterfly species [49-56]. Additional studies focusing on ommochrome and melanin candidate genes [57-61] increased the total of number of wing-pattern related genes to around 20 and provided some insight into the identity of potential downstream genes involved in pattern realization. The recent positional cloning of the *optix* color pattern gene [27] demonstrates the potential of forward genetics for identifying further genes. In this study we sought to accelerate gene discovery by moving beyond the candidate gene paradigm. Our work is the first large-scale expression assay for butterfly wing pattern genes. We have identified over 200 genes associated with color patterning, including several potential regulators of *optix* and a host of structural and pigmentation genes that have expression patterns that are correlated with adaptive color pattern variation in natural populations. These new data allow us to begin to understand the structure of the broader network of patterning and pigmentation genes in *Heliconius* and bring us closer to understanding the developmental genetic architecture of color pattern evolution*.*

### *Optix* and the color patterning gene network

Consistent with its role as a red color pattern switch gene [27], *optix* was the first transcript observed to show clear red-specific differential expression in our array data (Table 2, Figure 4). Furthermore, among the color-specific genes, *optix* had the longest persisting differential expression, lasting from Day 3 of the pupal stage through to ommochrome synthesis near the end of pupation (Figure 4, Table 2). Additional color-specific genes were expressed primarily during ommochrome and/or melanin pigment development. The long-term persistence of *optix* transcription accords well with its likely role as a selector gene [62], acting to reinforce positional fate through sustained expression rather than being part of a transient developmental cascade. Interestingly, out of the 51 genes that were expressed in association with *optix* there were no other obvious transcription factors or developmental signaling genes. Instead, the transcripts we identified largely represented genes involved in cuticle structure and pigment synthesis. Although our data are not exhaustive, they suggest that *optix* may play a relatively direct role in regulating scale and pigment development, as opposed to a more intermediate role in coordinating further downstream pattern formation processes.

The overall temporal sequence - *optix* expression followed by cuticle gene expression, which in turn was followed by pigment gene expression - is consistent with the progression of wing development, as scale maturation and sclerotization precedes the appearance of pigments (Figure 1B). Scales of different pigmentation also differ in cuticular fine structure, suggesting an interaction between cuticle formation and pigment synthesis [26]. Cuticle proteins compose a highly diverse gene family consisting of hundreds of genes involved in an extensive, yet poorly characterized, functional diversification [63-65]. The enrichment of this class of genes throughout the differentially expressed gene sets further suggests the importance of these genes in color pattern differentiation.

### Early prepatterning genes

Many of the genes differentially expressed across the proximal-distal wing axis were higher basally and involved structural genes, including extracellular matrix, cytoskeleton, and muscle genes. This may reflect potential differences in wing tissues at the basal hinge region versus the tip of the wing. Among these genes there was little evidence for expression differences being driven by developmental timing of scale development, as patterns involved consistent basal or apical differentiation across stages rather than evidence of delayed effects (as in Figure 3).

We found that early pupal genes expressed in association with proximal-distal wing pattern sections were enriched for morphogenesis and transcriptional regulation functions (Figures 2 and 3, Table 1), which was consistent with our expectation of finding genes involved in regulating development. Most of these genes have been previously recognized to play a role in *D. melanogaster* early wing axis formation, and several are worth highlighting as especially strong candidates for regulators of *optix*. In particular, orthologs of the transcription factors *zfh2, homothorax*, and *araucan* showed sustained proximal expression throughout pupal wing development, potentially suggesting selector gene roles for these molecules. *tiptop* also showed a strong association with the proximal section of the wing early in development, although its expression waned at Day 3 to Day 5, coincident with the onset of *optix* expression. As for the known functions of these candidates, in *D. melanogaster homothorax* is a homeodomain protein known to be involved in establishing proximal wing fate [66] and *zfh2* proximal expression plays a role in preventing distal fates [67]. *araucan* is a transcription factor primarily known for its role in *D. melanogaster* wing vein specification [68]. This is a particularly interesting new candidate gene because the rayed hindwing pattern develops relative to wing venation, with many of the rays positioned parallel to and halfway between the wing veins. *tiptop* is a selector gene involved in specifying positional identity in various insect appendages [69] and is known to interact developmentally with *homothorax* and *optix* in *D. melanogaster*[40]. *fourjointedvestigial*, and *distalless*, which showed a specific association with the distal tip of the pupal wing before and during *optix* differential expression, are known as distal appendage and/or wing determinants in *D. melanogaster*[ 70- 73]. *serratebowl*, and *wnt6* have less significant associations in our data, but are implicated in various aspects of positional specification in *D. melanogaster* wing development. Follow-up *in situ* hybridizations are needed to more rigorously assess the potential role of these genes in prepatterning.

### Ommochrome pigments: Enzyme regulation and novel transporters

Genes with color-specific differential expression almost always showed higher expression in red pattern elements (Table 2, Additional file 3). Perhaps unsurprisingly this pattern of upregulation encompassed many genes implicated in the synthesis of ommochromes, the class of pigments that imparts the red coloration in these butterflies and whose precursor, 3-OH-kynurenine, imparts the yellow pigmentation. However, some specific gene expression patterns we observed were unexpected and suggest that a revision of the current model of ommochrome synthesis in butterfly wings is required.

Most of what is currently known about the genetic basis of ommochrome synthesis comes from work with *D. melanogaster* eye mutants, and we have previously relied on this work to propose a model of how ommochromes might be produced in butterfly wings [58]. *D. melanogaster* ommochrome mutations tend to fall into three functional classes: transporters (e.g., *white, scarlet, karmoisin*), pigment synthesis enzymes (e.g., *cinnabar, vermilion, kf*), and granule formation proteins (e.g., *garnet, claret, ruby*). Previous work in *H. erato*[59] and *H. melpomene*[60] has shown that several of these ommochrome enzyme and transporter genes are expressed in *Heliconius* wings, and some of them, especially *cinnabar*, are strongly upregulated in red regions of the wing pattern. Beyond these gene expression associations, however, little is know about how similar ommochrome biosynthesis is between *D. melanogaster* eyes and butterfly wing scales. In particular, major questions remain regarding the specific precursors that are transported from the hemolymph into scale cells, whether there is anything analogous to pigment granules in scale cells, where precursor transporters are located in the scale-building cells, and what molecules might be active in later steps of ommochrome synthesis and stabilization.

In terms of the expression of enzyme genes, both *kf* and *cinnabar* were differentially expressed between color pattern morphs, but in different ways (Figure 5). *cinnabar* differential expression began after Day 5 and was higher in red and yellow regions in both ommochrome and melanin stages, with highest expression in yellow regions during the melanin stage. This expression pattern is consistent with the inferred role of *cinnabar* in the production of 3-OH-kynurenine, both as a precursor for red ommochromes and for deposition in the melanin stage as the yellow pigment. These results support local synthesis of 3-OH-kynurenine, in addition to the uptake of 3-OH-kynurenine from the hemolymph [59]. In contrast to *cinnabarkf* differential expression occurred during the ommochrome stage, where it was upregulated only in red patterns, with all tissues showing similarly high expression levels by the melanin stage. *vermilion,* which encodes the initial enzyme in the ommochrome pathway, has yielded inconsistent pattern of differential expression in previous studies, with evidence of higher expression within the red band in ommochrome stages by Reed et al. [59] and indication of differential expression only in late melanin stages by Ferguson & Jiggins [60]. In the cross-developmental analyses here there were no significant patterns of differential expression in *vermilion*; instead it showed high, ubiquitous expression early in pupal development (before *optix* expression), progressively dropping lower over time to barely detectable levels during the ommochrome and melanin stages. None of these enzyme genes showed any obvious pattern of spatial or temporal co-regulation, supporting the previous hypothesis that they are independently regulated [59], and that the control of timing of pigment synthesis may depend on the regulation of transporters of precursor metabolites.

Accordingly, some of the most interesting findings from our study relate to the expression of ommochrome precursor transporter genes. The ommochrome pigment transporter genes observed in *D. melanogaster* eyes - *whitescarlet*, and *karmoisin* - have uncertain roles in ommochrome synthesis in *Heliconius* wings. Ferguson and Jiggins [60] did not find *white* or *karmoisin* to be expressed at appreciable levels in *H. melpomene* wings but did find *scarlet* to be differentially expressed in the red mid-forewing band in ommochrome- and melanin-stages [60]. Our data showed that *white* and *scarlet* are expressed at low levels, with no significant color associations (Figure 4). Likewise, *karmoisin* was not even represented on our array because its expression levels were too low for its transcript to be identified through EST or 454 sequencing. In contrast to the results with *whitescarlet*, and *karmoisin*, we identified transcripts encoding two new ABC transporters potentially involved in pigmentation (*i.e.*, in the same functional class, but not the same subclass, as *white* and *scarlet*) and a poorly known monocarboxylate transporter (*i.e.*, in the same functional class as *karmoisin*) that were significantly upregulated at relatively high levels in red wing sections during ommochrome synthesis (Figure 5).

The pattern/transcript associations described above were made possible due to our ability to section forewing tissues along color pattern boundaries. In the hindwings, we lacked similar tissue-specific controls for comparisons between morphs, thus conclusions on hindwing-specific regulation are more tentative. Two differentially expressed genes in the hindwing of interest, *light* and *lightoid*, are thought to play a role in ommochrome pigment granule transport [45-47]. Other regulatory genes with potential color pattern function were differentially expressed only in hindwings (e.g., *ovoduskyseven in absentia*), however further work is needed to determine to what extent their expression differences are related to color pattern development.

Overall our ommochrome gene expression data call into question the applicability of the *D. melanogaster* eye model (Figure 5) to butterfly wing scales. The low or undetectable expression levels of *white*, *scarlet*, and *karmoisin* suggest that these transporters may play little or no role in pigment synthesis in butterfly wings. Conversely, our discovery of three novel color pattern-associated transporters implies that a significant portion of the ommochrome biosynthesis regulatory mechanism in butterfly wings may be quite different from that found in *D. melanogaster* eyes.

### Melanins: Color patterning by repression of pigment synthesis

Our results suggest that several melanin genes drive differences in pigmentation across color elements and forms. Among the known melanin genes represented on our array, *ebonyDat1*, and *yellow-d* were significantly differentially expressed between color pattern phenotypes (Figure 6). *Drosophila* has shown similar variation in genetic modifiers of melanism, with *yellowebony*, and *tan* variably implicated in driving melanic variation across species and natural populations [74-78]. Furthermore, our results suggest that variation of black *Heliconius* wing patterns may be achieved largely through the upregulation of melanin pigment repressors rather than reduced expression of melanin synthesis genes.

*ebony* differential expression was confined to the melanin-stage and showed upregulation in red tissues relative to other tissues, an expression pattern noted by Ferguson et al. [61] in several *Heliconius* species. This expression pattern is consistent with the known function of *ebony* in *D. melanogaster* where the ebony protein shunts dopa that would be used for making black or brown melanin to N-β-alanyl dopamine, thus resulting in a lighter yellow sclerotization [44]. The lack of dark pigmentation would thus enable more brilliant red coloration. While this is a simple and appealing model for red phenotypes, it does not apply to yellow phenotypes, which do not show *ebony* upregulation in those regions of the wing that are fated to be yellow. Thus, the question arises as to how yellow scale cells manage to repress dark melanization. In this regard, it was interesting to find that yellow-barred hindwings showed significantly higher expression of *Dat1* during the melanization stage. *Dat1* shunts dopamine, the precursor of black dopamine melanin, to N-acetyl dopamine, resulting in clear cuticle using NADA sclerotization [44]. This differential expression is thus an alternative way to eliminate the expression of dark melanins, and makes it possible for 3-OH-kynurenine to produce a vibrant yellow color. In addition to *ebony*, Ferguson et al. [61] found that *tan* is more highly expressed in black parts of the wing during mid to late melanin stages. Both *tan* and *pale* approach the upper end of gene expression across tissues, where it is difficult to detect differences using microarray technology. Although not differentially expressed, there is some indication in the early melanin stage that *tan* expression may be higher in black tissues.

*yellow-d* was the other known melanin gene we found differentially expressed between color patterns. It showed an expression profile very similar to *ebony*, with significant upregulation in red pattern elements during pigment synthesis stages. The yellow gene family has diversified into nine major lineages within insects [79]. The specific molecular functions of these yellow proteins are unknown, however several of the paralogs are known to play a role in pigmentation. *yellow* appears to have a conserved role in pigmentation, promoting melanization in *D. melanogaster*[ 74, 80], Coleoptera [81], and Lepidoptera [79,82]. In *B. moriyellow-d*[83] is also implicated in increasing melanization, while *yellow-e* is associated with white coloration in larvae [84]. Our finding that *yellow-d* is associated with a lack of melanization is therefore in contrast to previous results from *B. mori*[83]. Our results are in accord, however, with recent work in *H. melpomene* where upregulation of *yellow-d* and, to a lesser extent *yellow-h*, was observed in red tissues [79]. These results support the evolutionary labile functions of members of this gene family in pigmentation.

## Conclusions

Our relatively conservative analyses have identified a number of new genes associated with the development and variation of specific *Heliconius* wing pattern elements. In addition to independently recovering previously known wing patterning genes, we also identified a large number of novel associations that would likely never have been found by using a traditional gene-by-gene candidate approach. Beyond presenting a substantial roster of novel wing patterning genes, our study also provides a unique network-level glimpse into the genetic architecture of intraspecific phenotypic variation. There is speculation regarding the nature of so-called adaptive hotspot genes like *optix* that disproportionately drive phenotypic evolution across species [85]. Functional evolution of hotspot genes is expected to consist largely of *cis*-regulatory changes because they allow a highly context-specific fine-tuning of pleiotropic effects. The scale of pleiotropy encountered in natural cases of adaptive regulatory variation has rarely been assessed. We now have some insight into the number and types of elements that respond to adaptive allelic variation at the *optix* locus. This study has yielded many promising wing pattern candidate genes, has revised our understanding of prepatterning and pigment regulation in *Heliconius,* and set an important foundation for understanding the genetic interactions that regulate the remarkable wing pattern diversity seen in butterflies.

## Competing interests

The authors declare that they have no competing interests.

## Authors’ contributions

WOM, HFN, and HMH developed and organized the hindwing project. HMH collected tissues and microarray data and performed analyses. RDR, WOM, HFN, and RP developed and organized the forewing project. RP and RDR collected tissues and RP and CW collected microarray data. RP, MR, and CW assisted in analysis. AP performed the transcriptome assembly. WOM and RDR provided advisory and financial support. HMH wrote the paper with strong input from RDR. All authors provided editorial support and approved the manuscript.

## Supplementary Material

Additional file 1 ** Summary of the genes known to interact with *optix* in* D. melanogaster.*** Genes present and differentially expressed in our study are indicated. Click here for file

Additional file 2 ** Full list of all genes differentially expressed along the proximal-distal axis of the forewing.** Functional clusters are based on results from gene functional classification analysis in DAVID (Table 1). Abbreviations and style follow Figure 3. Click here for file

Additional file 3 ** Expression patterns and transcript IDs for genes in Table** 2**and for additional differentially expressed pigment-related hindwing genes.** Formatting of the first two columns follows Table 2. The heat map follows formatting of Figure 3, except that hindwing expression is shown (amp = *H. e. amphitrite*, ema = *H. e. emma*, fav = *H. e. favorinus*) and the morphs are colored respective to their color phenotype. Click here for file
